# Scholar Metrics Scraper (SMS): automated retrieval of citation and author data

**DOI:** 10.3389/frma.2024.1335454

**Published:** 2024-02-22

**Authors:** Yutong Cao, Nicole A. Cheung, Dean Giustini, Jeffrey LeDue, Timothy H. Murphy

**Affiliations:** ^1^Department of Psychiatry, Faculty of Medicine, University of British Columbia, Vancouver, BC, Canada; ^2^Djavad Mowafaghian Centre for Brain Health, University of British Columbia, Vancouver, BC, Canada; ^3^Biomedical Branch Library, University of British Columbia, Vancouver, BC, Canada

**Keywords:** Google Scholar, Python, bibliometrics, citation metrics, research impact, automation

## Abstract

Academic departments, research clusters and evaluators analyze author and citation data to measure research impact and to support strategic planning. We created Scholar Metrics Scraper (SMS) to automate the retrieval of bibliometric data for a group of researchers. The project contains Jupyter notebooks that take a list of researchers as an input and exports a CSV file of citation metrics from Google Scholar (GS) to visualize the group's impact and collaboration. A series of graph outputs are also available. SMS is an open solution for automating the retrieval and visualization of citation data.

## 1 Introduction

The most common metrics used to examine productivity and influence in science are linked to a researcher's publications, and the number of times they are cited by others (Schreiber and Giustini, 2019). The h-index combines two key metrics, publication and citation counts, into a single number that can be used to evaluate a researcher's impact (Hirsch, 2005). Patterns arising from these citation metrics support a range of activities from identifying collaborative performance and research trends to strategic planning. Tenure and promotion committees, granting agencies and administrators spend a lot of time evaluating the impact of author publications (De Silva and Vance, 2017). Co-authorship analysis can reveal collaborations between authors and provide insights about interdisciplinary research. In reviewing groups of researchers, understanding relationships and patterns of intra-group collaboration can be used to create management strategies and identify key authors within academic networks.

Key citation indexes are used to perform bibliometric analysis and to extract accurate citation data. Google Scholar (GS), OpenAlex (Priem et al., 2022), OpenAIRE Graph (Manghi et al., 2023), Scopus and the Web of Science (WoS) aggregate scholarly publications and provide access to bibliometric data such as citation counts and the h-indexes of authors. Moral-Muñoz et al. (2020) provides a more detailed overview of various bibliometric tools for bibliometric scraping and visualization. However, while Scopus and WoS require subscriptions to access and index peer-reviewed publications (Halevi et al., 2017), they do not include all scholarly pre-prints or theses. Conversely, OpenAlex and OpenAIRE Graph are open search tools covering a wide swath of publications and scholarly outputs on the web, including those outside of traditional scholarly journals. Similarly, GS compiles publications from across the web allowing access to a greater range of documents (Halevi et al., 2017; Martín-Martín et al., 2021). We focus here on automating aspects of data-retrieval and visualization from GS as it has been a publicly-accessible database since 2004, with an established user base among academics.

We present Scholar Metrics Scraper (SMS), an open-source tool that uses the *scholarly* package to automate data extraction from GS that provides flexible methods of visualization using chord diagrams to assess interactions between a list of individuals. The project uses Jupyter notebooks to provide step-by-step instructions for modification, and no programming experience is needed. SMS users can upload author names to retrieve data types supported by the *scholarly* Python package which are organized into a table. This table is used to create figures to track a research group's collective scholarly output, identify key members, and visualize intra-group collaboration. To illustrate the SMS tool, we provide use cases for citation data obtained from GS, Scopus, and OpenAlex for individuals within the Dynamic Brain Circuits in Health and Disease Research Excellence Cluster at the University of British Columbia. SMS visualizes collaboration within user-adjustable chord diagram formats.

## 2 Related works

Many existing research analysis tools can process data downloaded from Scopus and WoS (Moral-Muñoz et al., 2020), but similar tools are not available for GS, OpenAlex, and OpenAIRE Graph. For data scraping, Elsevier's SciVal and Clarivate's InCites are proprietary tools used to access author metrics from Scopus and WoS, respectively (Bornmann and Leydesdorff, 2012; Dresbeck, 2015; InCites Benchmarking and Analytics, 2023), but they require a subscription to access. Although third party tools such as Bibliometrix and Publish or Perish retrieve citation data from several sources including GS (Harzing, 2007; Aria and Cuccurullo, 2017), they are not designed for groups of researchers. To gain access to GS bibliographic data, Python packages such as *scholarly* provide built-in methods to retrieve citation data and individual author profiles from GS (Cholewiak et al., 2023); however, they require coding experience to implement fully. OpenAlex has interactive features to export search results (Priem et al., 2022), but extracting data for multiple researchers requires knowledge of API calls and JSON object manipulation.

VOSViewer is a tool created for both bibliometric data collection and visualization (Van Eck and Waltman, 2010). VOSViewer can handle data output from Scopus and WoS, and has additional built-in functionality for data scraping and network construction from a variety of both public and private tools such as Crossref, WikiData, OpenAlex, and SemanticScholar. However, GS integration with VOSViewer is not available. When downloading data using APIs, VOSViewer lacks grouped co-authorship data functionalities. While it can create a co-authorship map of all co-authors from a single author's list of publications, it does not have an option to scrape for co-authorship patterns between a list of specified individuals. Thus, specific methods are needed to gather bibliometric data from GS for groups of authors in order to evaluate their contributions and collaborations.

## 3 Methods

**Figure 1:**
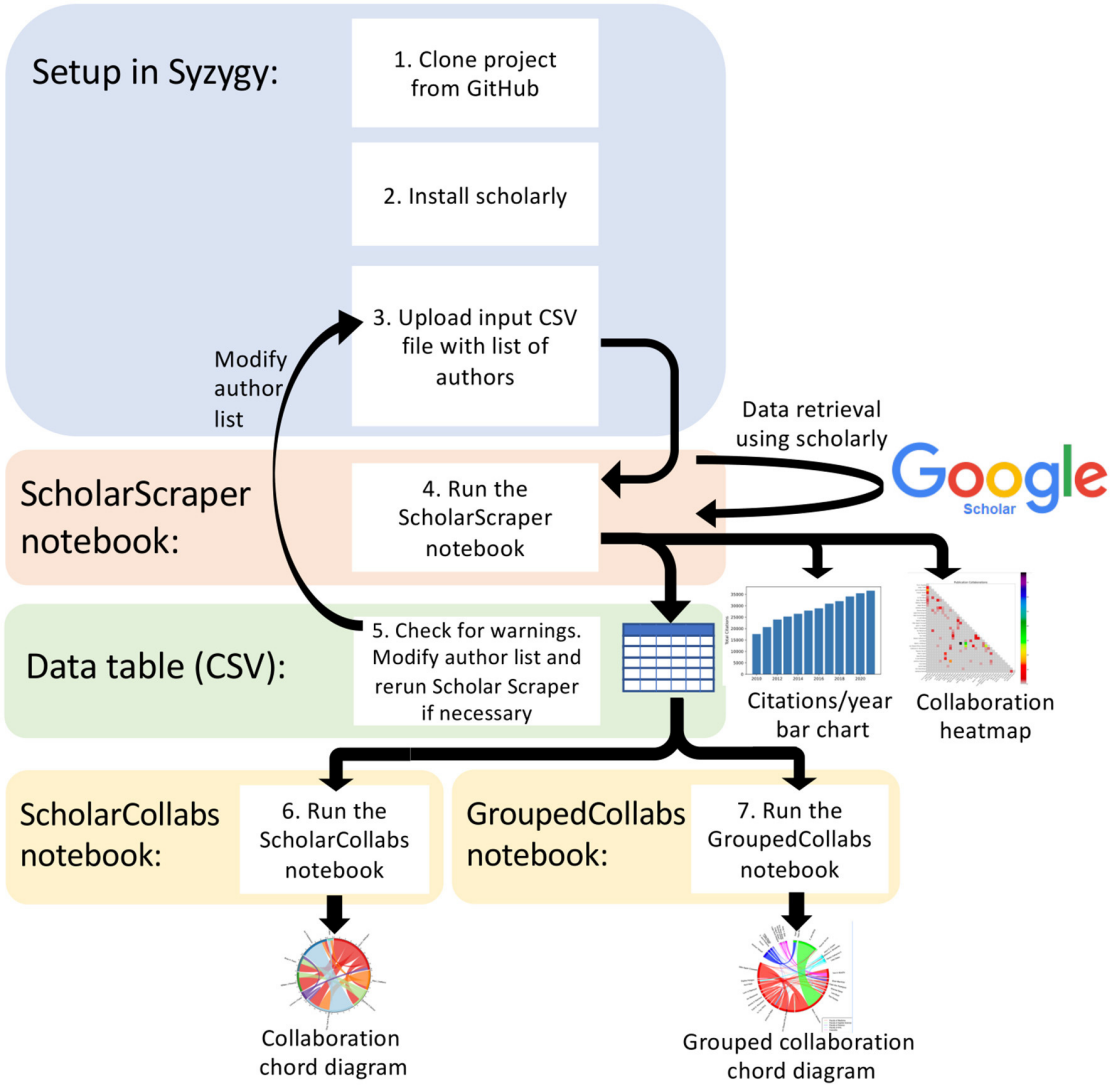
Scholar Metrics Scraper workflow diagram including the architecture and major steps.

Using interactive Jupyter notebooks and previously published components that interact with the GS database (Cholewiak et al., 2023) we have created a tool called Scholar Metrics Scraper (SMS). Details on how to construct and employ this software with GS, Scopus and other outputs are outlined below. A definition of terms has been provided in Supplementary Table 1. Speciality notebooks are developed which gather citation data (ScholarScraper notebook; Figure 1, step 4), and visualize co-author collaboration data (ScholarCollabs notebook and GroupedCollabs notebook; Figure 1, steps 6–7). The software is platform independent and uses the Python (3.10.0) and R (4.2.2) coding languages.

We compiled a list of 47 members from the University of British Columbia Dynamic Brain Circuits Cluster to demonstrate our tool (Supplementary Table 2). 10 out of 47 member profiles are retrieved using GS IDs.

### 3.1 Software architecture

Our SMS project is available on GitHub at https://github.com/ubcbraincircuits/scholar_metrics_scraper. Step-by-step instructions for Syzygy setup are provided on GitHub at https://bit.ly/3u0OMAn. Users are instructed to open Syzygy, which provides free online access to Jupyter (Lamoureux, 2020), and log in with their institution or Gmail account. To set up a project in Syzygy, users can follow instructions to clone the project into their Jupyter directory (Figure 1, step 1) and install the *scholarly* Python package (Figure 1, step 2). Users create a CSV file with author names or GS identifiers (GS IDs) in a single column (see Appendix A) and upload the file to their Jupyter directory (Figure 1; step 3). A GS ID is alphanumeric and embedded within the GS profile's uniform resource locator (URL). For example, where an author's profile URL (Dr. Tim H. Murphy) is “https://scholar.google.ca/citations?user=qJjM8hkAAAAJ&hl=en”, the corresponding GS ID is qJjM8hkAAAAJ.

### 3.2 ScholarScraper notebook

**Figure 2:**
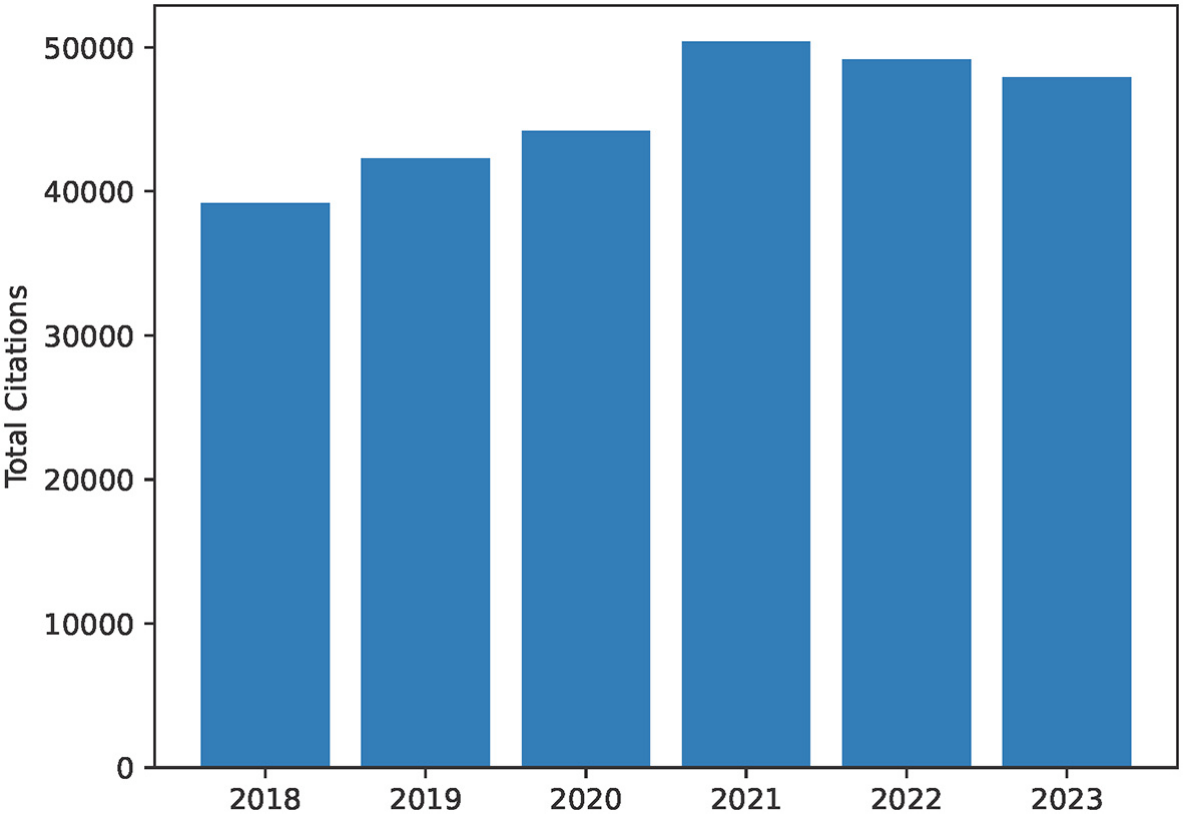
Total citations per year as a bar chart from 2018 to 2023. This includes citations for publications by all authors in the author list for the University of British Columbia Dynamic Brain Circuits in Health and Disease Research Excellence Cluster. This diagram is created as output by the ScholarScraper notebook, and the range of years can be easily modified. The graph reflects the total citations for the cluster and thus may contain duplicates within collaborative projects.

**Figure 3:**
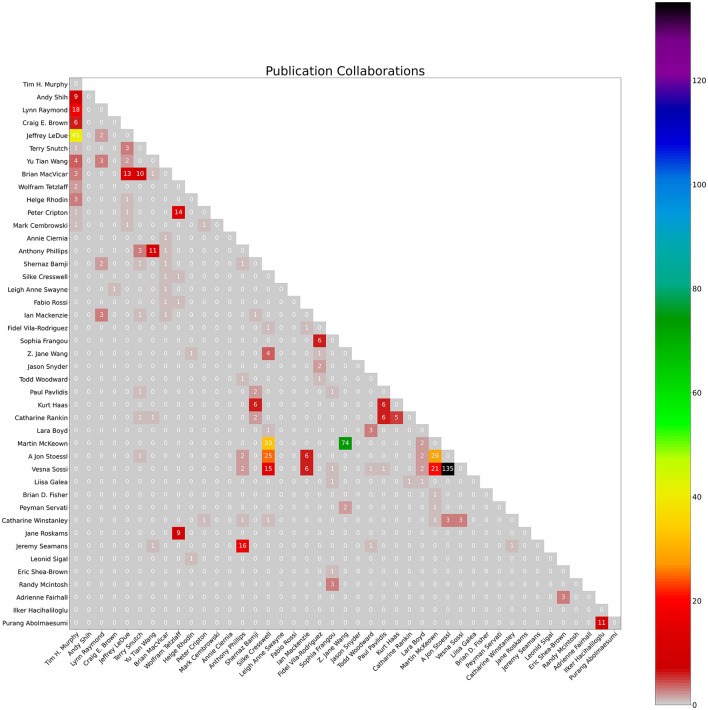
Count of collaborations heatmap. The numbers represent the count of publication collaborations between co-authors as of January 4th, 2024.

The ScholarScraper notebook (Figure 1, step 4) contains Python code cells with step-by-step instructions to load in the author list, retrieve data from GS, and produce a table (Supplementary Table 4) and two figures (Figures 2, 3). Users make minor modifications to the code to load in their author list and provide a list of institution names with which the authors are collectively affiliated. The ScholarScraper notebook iterates over the supplied author list to retrieve author profile data from GS by either using *scholarly*'s “search_author” function or “search_author_id” function, prioritizing the GS ID for author retrieval if it was provided in the “GSID” column. To inform users if the retrieval is unsuccessful or if there may be author name ambiguity, the output table includes a warning column (Supplementary Table 4).

Only researchers with GS profiles will be retrieved. Scholars can set up their own GS profiles by manually adding their biographical information, expertise and keywords, and curating their publications (Thoma and Chan, 2019). If *scholarly* does not find an existing GS profile under the author's name, the ScholarScraper notebook will output an error resulting in a blank row in the output table, and a warning will be given that no profile was found.

*Scholarly* will find the author profile with the closest match, but may retrieve another author's profile with a similar name. For example, if “John Doe” is on the author list but there are other “John Does” at different institutions, *scholarly* will return the profile with the highest GS page rank (in the order of which profiles appear according to the GS profile search results), which may not be the desired profile. In another case, if “John Doe” does not have a GS profile, the *scholarly* search may look for “J Doe” and subsequently return “Jane Doe”. To address this issue, the notebook checks each author's affiliation (retrieved from GS) against the affiliations list as provided by the user in the code. When none of the strings match the author's affiliation, a message that the affiliation did not match will appear in this column.

Users should manually compare the output data file's “Name” column with the “GS Profile Name” column to check that the correct user was selected. As necessary, users can use the output data as a reference for modifying the author list (either supplying the researcher's GS ID in the author list “GSID” column or including more specific information such as a middle initial in the author list “Name” column) to ensure the correct author profile is retrieved, and run the notebook again for corrected results (Figure 1, step 5). Any profiles retrieved with the GS ID will not display a warning, even when the affiliation name does not match, as it is assumed that the profile is correctly attributed through manual lookup. This narrows down the amount of required manual searching, as the user only needs to focus on validating the profiles marked with a warning.

The output data, stored as a CSV file, uses *scholarly*'s “fill” function to directly retrieve data such as publication and citation counts, h-index, publication titles (Supplementary Table 4). The notebook is flexible and can be modified to retrieve any additional GS author profile information available to *scholarly* by modifying the “row” dictionary data structure and the “keys” variable. Co-author information in the output data file (Supplementary Table 5) is created by retrieving the title of every publication created for every author, and grouping together authors who have listed the same article name on their profile. Authors with the same publication titles are then added to each others' co-author dictionaries. This process is necessary due to the lack of co-author metadata that can be retrieved from GS.

The notebook also produces a bar graph of cumulative yearly citations of the group over the past 5 years, and a heatmap visualizing co-author collaborations in a matrix. The notebook takes each author's citations each year over time and totals them to create a bar chart of total citations per year for the group. When two authors in the group have a common publication as collaborators, citations for this publication will be double-counted. Therefore, yearly citation totals represented by the bar chart may overcount the group's citations. The heatmap uses the co-author data of the earlier co-author process (described above). These graphs are created using the Python *matplotlib* package (Caswell et al., 2023) using output data from the earlier scraping process.

### 3.3 ScholarCollabs notebook and GroupedCollabs notebook

**Figure 4:**
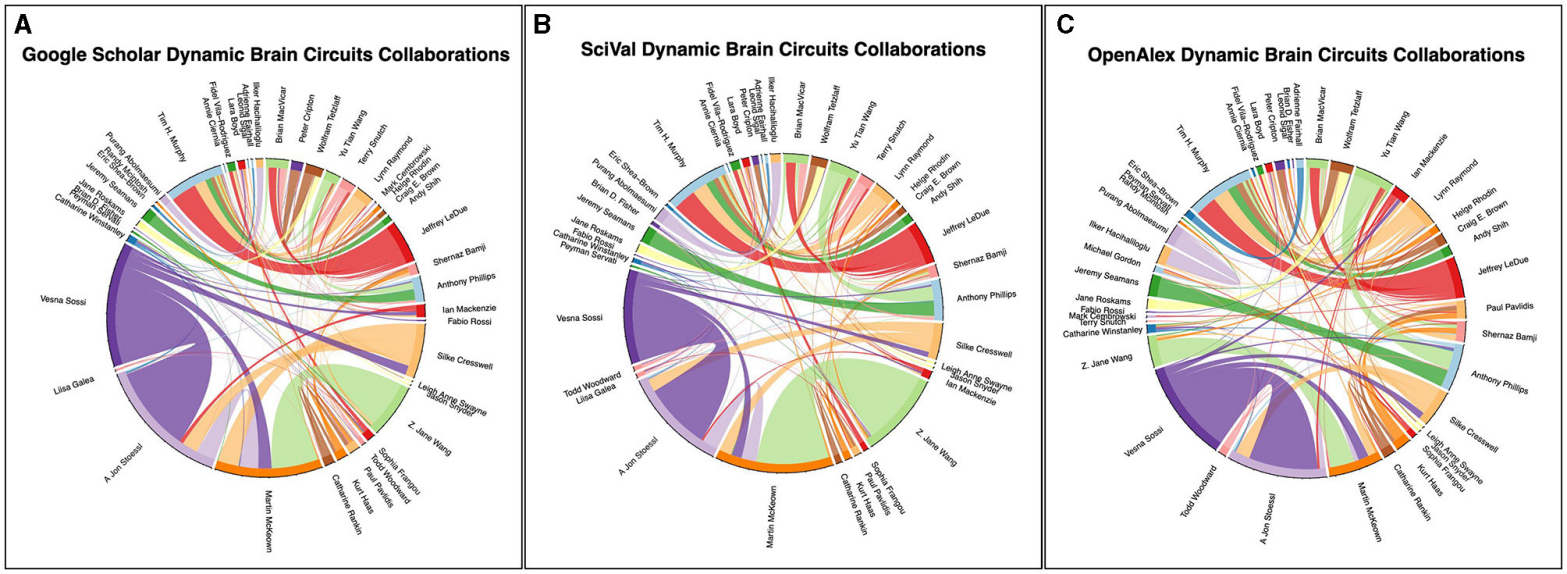
Collaboration chord diagram produced by the ScholarCollabs notebook. Links are weighted by the number of collaborations. The ScholarCollabs notebook creates this collaboration diagram based off of the Name and co-authors columns (Supplementary Tables 5, 6) of the author data table that is created as output by the ScholarScraper notebook. **(A)** represents data from the Google Scholar database, **(B)** represents data from the SciVal database, and **(C)** represents data from the OpenAlex database as of January 4th, 2024.

**Figure 5:**
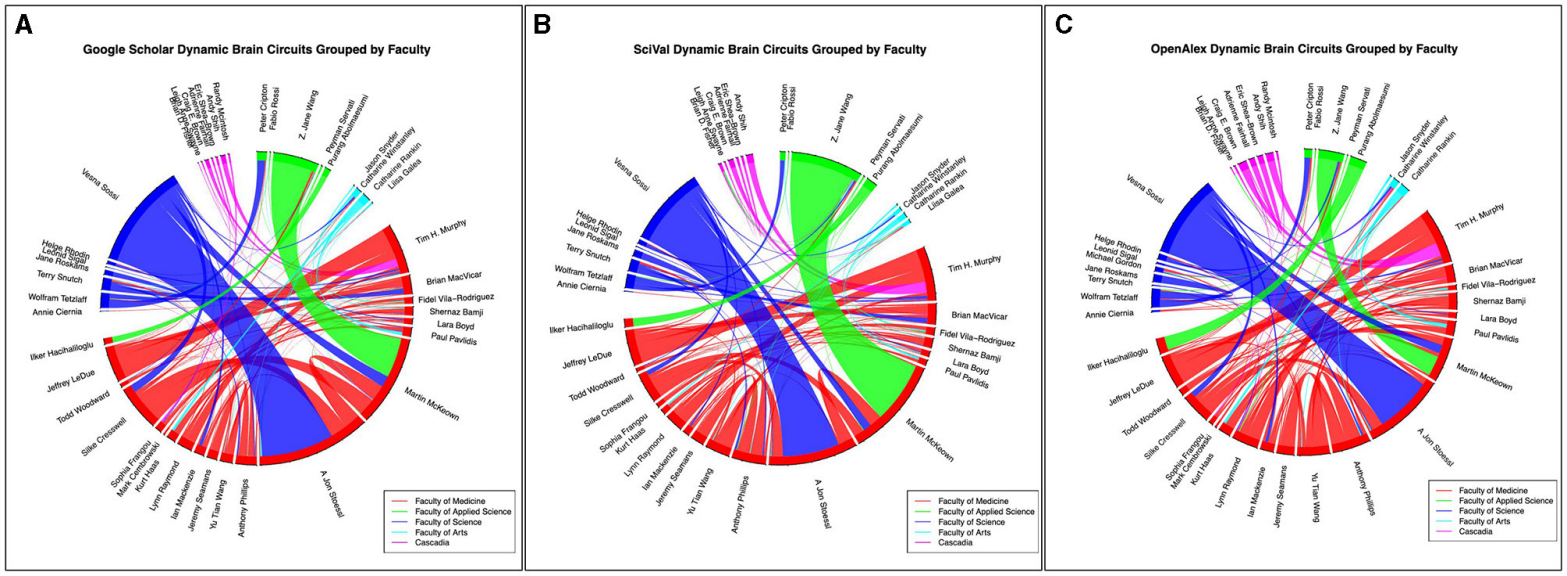
Grouped collaboration chord diagram created by the GroupedCollabs notebook based on faculty or institution. Links are weighted by the number of collaborations. **(A)** represents data from the Google Scholar database, **(B)** represents data from the SciVal database, and **(C)** represents data from the OpenAlex database as of January 4th, 2024.

The ScholarCollabs and GroupedCollabs notebooks (Figure 1, steps 6–7) are written in the R programming language to create collaboration diagrams with step-by-step instructions. The ScholarScraper output CSV file, particularly the columns concerning author names, their co-authors, and the number of publications they share with each co-author, is used by both ScholarCollabs and GroupedCollabs. These notebooks turn the co-authorship data into an adjacency list, which is a list of the edges that represents the whole co-authorship network. We use the *circlize* R package (Gu et al., 2014) to draw the graph. Users can follow instructions to modify the file name objects, title, and indicate if they want a weighted or unweighted diagram. This creates a chord diagram which visualizes co-authorships as links between authors (Figure 4). The diagram is saved in the Jupyter directory as a PDF which can be downloaded locally. The GroupedCollabs notebook produces a diagram with authors grouped into subgroups (e.g., faculty, research area, etc.) (Figure 5). It works similarly to ScholarCollabs but requires an additional CSV file which contains the group which each author belongs to Supplementary Table 3.

### 3.4 Co-authorship matrix and social network analysis

To demonstrate possible applications of the SMS tool, we implemented this workflow for data collection with the proprietary Scopus database using SciVal API calls and with the OpenAlex database using the OpenAlex API.

SciVal profiles and associated IDs of Dynamic Brain Circuits Cluster members were retrieved from a SciVal account used for quantifying cluster and research group output and reporting on activities affiliated with the Djavad Mowafaghian Center for Brain Health. The “AuthorRetrieval” function in the *pybliometrics* Python package was used to collect publication information (Rose and Kitchin, 2019). SciVal bibliometrics were obtained in the same framework as the ScholarScraper notebook by substituting the *scholarly* functions with the *pybliometrics* functions.

OpenAlex IDs were collected by querying the OpenAlex “authors” API with the author's name directly from the author list provided to GS. Author IDs were supplied if they have been previously associated with institutions of interest (University of British Columbia, Simon Fraser University, 'University of Victoria, or University of Washington) and also have been published in certain subjects of interest ('Biology', 'Medicine', 'Computer science', or 'Psychology'). This process is demonstrated in the notebook *OpenAlex_find_profiles.ipynb* in the “Supplemental Data/OpenAlex Documents” subdirectory of our GitHub repository. To collect OpenAlex bibliometric indicators, we used the same framework as SMS and extracted the desired variables from the JSON response in place of the *scholarly* functions.

As GS and Scopus are not equivalent databases in size and produce different citation counts, we validated the different titles obtained from GS and SciVal to reveal numbers of publications in both, and numbers of unique publications in each. Even though titles from both GS and SciVal referred to the same publication, titles were normalized for comparison. For example, the same publication containing quotation marks might be formatted using straight quotes (””) on one database and typographic curly quotes (“”) on the other. To normalize the title formatting differences, we remove superscript and subscript tags, remove all non-alphanumeric symbols, and standardize to lowercase letters. This allows us to compare the co-authored documents gathered from GS and SciVal to determine similarities and differences in both databases.

We conducted a social network analysis using the co-authorship matrices obtained from the data table (Figure 1, step 5) for both GS and SciVal data as an example of applications with this data. Each value in this matrix is then inverted: the more papers the co-authors publish together, the smaller the edge weight is between those author nodes. Using the notebook, a social network can be created with authors as the nodes and number of co-authored publications as the edges. This collaborative co-authorship network can reveal cohesiveness of the group and identify individuals who are well positioned and best connected between all members.

Using the “betweenness_wei” function of the *bctpy* Python package [bctpy (v0.6.1): Brain Connectivity Toolbox for Python, 2023], we calculated measures of betweenness centrality on the co-authorship matrix. We apply a weighted version of the betweenness centrality measure, which considers the edge weights, or number of publications, that two authors have co-authored together. We do this by first inverting all the publication counts– for example, if author A and B co-authored 3 publications, the weight of their edge is set as 1/3. Betweenness centrality is based on the number of shortest paths between two nodes. A path is a set of edges that connect two nodes. Sometimes the shortest path between one node and another relies on intermediate nodes along the way. The betweenness centrality of a node is a reflection of how many times that node lies on the shortest path between pairs of other nodes. Visualization of these results are done using the Python *matplotlib* package (Caswell et al., 2023). Code and data used to create the output graphs are in the “Additional Data/co-authorship Analysis” folder on the GitHub repository.

## 4 Results

### 4.1 ScholarScraper notebook

Graphical outputs include the total citations per year bar chart (Figure 2) and the heatmap of co-author collaborations (Figure 3) from members of the University of British Columbia Dynamic Brain Circuits Cluster. The ScholarScraper notebook found 484 co-authored publications on GS and 374 co-authored publications on SciVal. Of these publications, the matching method found 177 titles exclusive to GS, 67 exclusive to SciVal, and 307 titles shared by both. It is expected that GS will contain more unique titles (theses, presentations, etc) than SciVal. Due to the presence of additional words in SciVal titles (such as versions of the corrections or the DOI) that could not be automatically filtered out, there may be more matching publications than those detected using our method. Publication titles with associated co-authors can be found on the GitHub repository in the folder titled “Publication Comparison” in the “Supplemental Data” directory.

Over the process of co-author publication validation, we discovered at least 3 titles containing text irregularities or typos. We found that Sophia Frangou and Randy McIntosh's GS profiles contained multiple PDFs with the header “A Journal Devoted to Functional Neuroanatomy and Neuroimaging” that was sometimes misspelled with “Functional” or “Funconal”. This resulted in three instances of collaboration, when they should be represented as one. Another error occurs when GS is unable to locate the actual title of the publication within a PDF, and accidentally uses a header or journal name for the title instead. Paul Pavlidis, Sophia Frangou, and Vesna Sossi's GS profiles each contain at least one PDF with an “Accepted Manuscript” header which GS misidentifies as “ÔØ Å ÒÙ × Ö ÔØ”. They are grouped together as co-authors using the title-matching algorithm even though the actual publications titles are not the same. Co-authored GS publication scores may be slightly inflated without manual curation of the records with artifacts in the titles.

### 4.2 ScholarCollabs notebook and GroupedCollabs notebook

The ScholarCollabs and GroupedCollabs notebooks produce a collaborative chord diagram (Figure 4A) and a grouped chord diagram (Figure 5A) respectively. These notebooks are flexible with data obtained from other citation data sources, such as from the Scopus database through SciVal (results shown in Figures 4B, 5B). Ensure the data from the alternate source is stored in a dictionary data structure format with co-author name as a key and number of co-authored publications as a value. The table of SciVal data that is used by the ScholarCollabs and GroupedCollabs notebooks is included in the Supplementary material.

### 4.3 Social network analysis

**Figure 6:**
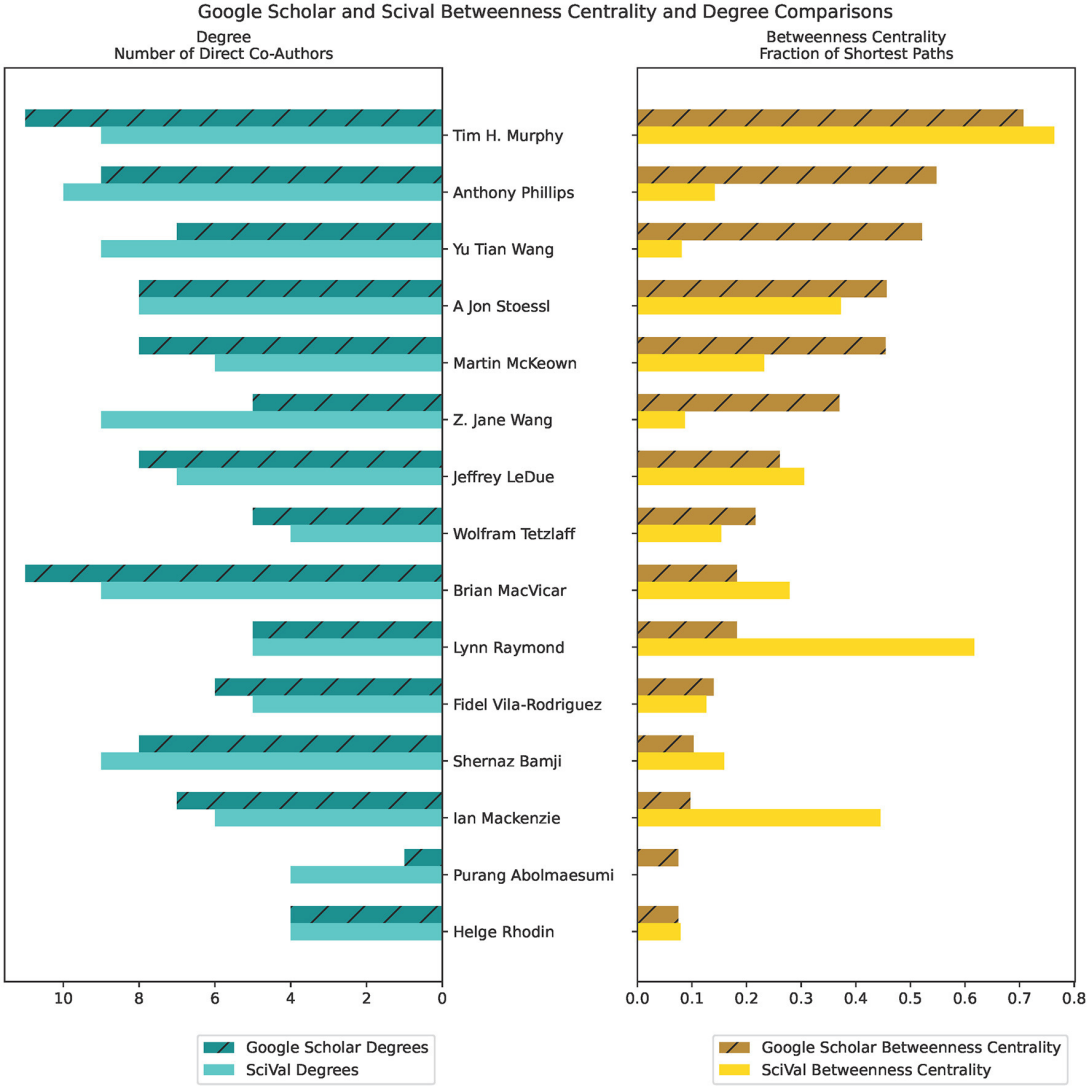
Betweenness Centrality and Degree measures of comparison between Google Scholar and SciVal, using co-author citation matrices created from the ScholarScraper notebook. Displays the first 15 authors in descending order of Google Scholar Betweenness Centrality value.

We include an example of co-authorship betweenness centrality comparisons between data extracted from GS and SciVal (Figure 6). The higher the number of shared publications, the higher the instances of collaboration and mutual production of knowledge. The bar graph on the left displays the node's degrees, or the number of direct co-authors that the author has worked with, while the bar graph on the right displays the betweenness centrality (higher values indicate more influence on network nodes) for that author. Authors are listed in descending order from the highest degree centrality measurement.

## 5 Discussion

Bibliometric studies have investigated the strengths and limitations of a range of metrics as measures of research impact (Agarwal et al., 2016). Studies use bibliometric data to highlight the research topics in a field with the most citations, publication topic trends, and strength of collaboration networks between researchers (Buchan et al., 2016; Yeung et al., 2017; Devos and Ménard, 2020). However, citation metrics alone may favor some areas of research over others, and single metrics such as the h-index are biased toward older researchers with more publications (Agarwal et al., 2016; Buchan et al., 2016). Agarwal et al. recommends that funding and recruitment decisions should incorporate qualitative peer review alongside multiple bibliometric indicators according to the goals of the evaluation. The biases of each indicator should be taken into consideration. Our tool helps by collecting bibliometric data and a range of indicators for a group of investigators in an automated, transparent manner.

### 5.1 ScholarScraper notebook

GS does not provide a way to extract data from its site natively, unlike other data sources that provide APIs for users to request information. GS profiles may include lists of co-authors, but these lists must be added manually, and may not be representative of all collaborators with whom the author has worked (Chen et al., 2017). Extracting co-author data from GS is possible using *scholarly* (Cholewiak et al., 2023) but not guaranteed to succeed due to incomplete metadata. *Scholarly* can retrieve a list of co-authors by scraping the extended information of a single publication. However, as authors can have hundreds of documents, looking up every publication for every author can cause heavy traffic to GS servers and lead to the user's IP address getting temporarily blocked (Cholewiak et al., 2023). Instead, we match co-authors based on the titles of shared publications: when the exact title of a publication appears in two or more authors' publication lists, they will be added to each other's co-author list. This prevents any double-counting of duplicated titles. However, since publications on GS are automatically scraped from many online sources, the title can be mistaken or misidentified by machine. This will result in co-author pairings being over-counted, as the same publication's title may be listed separately and misspelled in each instance.

To address this problem, future functionalities may be implemented to identify and remove suspicious titles, or use more stringent publication-matching requirements than solely the title for co-authorship. Though GS does not contain any accessible DOI information associated with their publications, it could be possible to obtain a document's metadata using external retrieval sources for matching purposes. We have found that this over-counting happens for 4 out of the 484 co-authored papers on GS and does not appreciably change the overall structure of the network.

We note that IP address blocking is less of a concern for OpenAlex and OpenAIRE Graph. Both provide API calls integrated directly into their database, which have limits listed (Priem et al., 2022; Manghi et al., 2023). Additionally, OpenAlex provides publication type and co-author metadata which is not available from GS.

### 5.2 Social network analysis

Co-author data obtained from the notebook allows for more insights into the group's activities by providing data for co-authorship analysis. As such, co-authorship networks are useful in investigating the exchange and production of knowledge and developing strategies and targets in research group management. High betweenness centrality is an indicator of an author who links together researchers with different research interests, and may reveal their interdisciplinary nature (Yan et al., 2010).

Differences between the Scopus and GS betweenness centrality measures we observe may be due to differences in the publications associated with author profiles in their respective databases. This can be seen in the different chord widths for co-authors between the GS, SciVal, and OpenAlex's chord diagrams in Figures 4, 5—each source has a different number of publications associated with the author profiles. GS tends to have a higher number of publications due to the inclusion of a larger variety of sources, as stated in the introduction. Some authors have separate profiles with publications that are not merged together in GS, and the profile with highest citation measures was selected. This leaves out potential co-authored publications, as the author's publications are split between each profile. Additionally, co-author analysis may not take into account differences in authors' career stages and may be biased toward researchers who conduct interdisciplinary work. Statistical measures and other modes of co-authorship network investigation may be conducted in the future to draw from the underlying distributions to examine claims about different databases.

Future avenues of exploration with SMS could involve comparisons of publication coverage and network diagrams between the OpenAIRE Graph, OpenAlex, and GS or Scopus databases.

### 5.3 Scholar Metrics Scraper practical usage

In our experience, researchers who want to find bibliometric data (for strategic planning purposes, evaluating researchers for promotion, or applying for grants, for example) do so by manually searching for each researcher and tracking data, or by using commercial packages such as SciVal. Previous studies on co-authorship and research group performance using GS have required the development of independent web-scraping methods (Chen et al., 2017), or manual addition of publications for each researcher in a research group (Thoma and Chan, 2019). Manual methods are time consuming and error-prone. By addressing the need for automating metrics gathering, from GS in particular, we sought to ensure accessibility and reproducibility in the spirit of open science. SMS is open source, does not require coding experience, and comes with a guide to setup and run the notebooks. To date, the SMS has been used by five Research Excellence Clusters at the University of British Columbia to assess collaborative activities and productivity.

## 6 Conclusion

SMS automates the process of retrieving a comprehensive range of bibliometric data from a list of authors. SMS extends existing tools for GS by implementing functionality for collecting co-authorship data. SMS is built on Jupyter Notebooks in both R and Python, featuring written instructions and executable code snippets for each step of the process for users to easily navigate and customize. SMS provides a framework to locate researcher profiles and extract information from bibliometric databases using API calls, allowing users to interact with Scopus, OpenAlex (Priem et al., 2022), and OpenAIRE Graph (Manghi et al., 2023) directly. SMS is an open-source tool, accessible to those without coding backgrounds, and customizable to the specific needs of other projects and research groups. Scholar Metrics Scraper allows for fast, simple, and flexible data collection and visualization, and provides co-author counts to assess collaboration.

## Data availability statement

The original contributions presented in the study are included in the article/Supplementary material, further inquiries can be directed to the corresponding author.

## Author contributions

YC: Writing—original draft, Writing—review & editing, Data curation, Methodology, Software. NC: Data curation, Methodology, Software, Writing—original draft, Writing—review & editing. DG: Writing—review & editing. JL: Writing—review & editing, Conceptualization, Methodology, Software, Supervision, Funding acquisition. TM: Writing—review & editing, Conceptualization, Supervision, Writing—original draft, Funding acquisition.
