# Supplementary material for: Scholar Metrics Scraper (SMS): automated retrieval of citation and author data
Source: Front Res Metr Anal. 2024 Feb 22;9:1335454. doi: 10.3389/frma.2024.1335454 (PMC10917922; doi:10.3389/frma.2024.1335454)
Supplement: Supplementary file 1 [file Table_1.DOCX]

***Supplementary Material***

# Supplementary Tables

| Scholar Metrics Scraper (SMS) | Our project contains the notebooks ScholarScraper, ScholarCollabs, and GroupedCollabs. It is available on GitHub at <https://github.com/ubcbraincircuits/scholar_metrics_scraper>. |
| --- | --- |
| ScholarScraper notebook | Contained in SMS as ScholarScraper.ipynb, it takes a list of authors as input, retrieves data from Google Scholar for each, and puts them into a table in an output CSV file. It also creates a citations/year bar chart and a collaboration heatmap as output. |
| ScholarCollabs notebook | Contained in SMS as ScholarCollabs.ipynb it takes the data table created by the ScholarScraper notebook and creates a collaboration chord diagram to visualize links between co-authors. |
| GroupedCollabs notebook | Contained in SMS as GroupedCollabs.ipynb it works similarly to ScholarCollabs, but groups authors into subgroups to create a grouped collaboration chord diagram. |
| Google Scholar (GS) <http://scholar.google.com> | The web search engine is used to search for scholarly literature and provides access to citation and author metrics. Open and publicly-accessible, GS is the largest scholarly search engine on the web providing access to more than 300 million references. |
| scholarly | A Python package that provides built-in methods to retrieve author data from GS for a given researcher [(Cholewiak et al., 2021)](https://paperpile.com/c/8QdBIq/jTip) |

**Supplementary Table 1.** Definition of terms

| **Name** | **GSID** |
| --- | --- |
| Tim H. Murphy |  |
| Annie Ciernia |  |
| Brian MacVicar |  |
| Fidel Vila-Rodriguez |  |
| Shernaz Bamji |  |
| Lara Boyd |  |
| Paul Pavlidis |  |
| Martin McKeown |  |
| A Jon Stoessl |  |
| Peter Cripton |  |
| Jason Snyder |  |
| Wolfram Tetzlaff | HgUvr-IAAAAJ |
| Anthony Phillips |  |
| Catharine Winstanley |  |
| Yu Tian Wang |  |
| Jeremy Seamans |  |
| Terry Snutch | mTkQFQ8AAAAJ |
| Ian Mackenzie |  |
| Lynn Raymond |  |
| Kurt Haas |  |
| Mark Cembrowski |  |
| Fabio Rossi |  |
| Jane Roskams |  |
| Catharine Rankin |  |
| Michael Gordon |  |
| Leonid Sigal |  |
| Z. Jane Wang | W75uTm8AAAAJ |
| Peyman Servati |  |
| Liisa Galea | Q--hI3oAAAAJ |
| Sophia Frangou | iRcTmwkAAAAJ |
| Silke Cresswell |  |
| Helge Rhodin |  |
| Manu Madhav |  |
| Brian D. Fisher |  |
| Leigh Anne Swayne |  |
| Craig E. Brown |  |
| Adrienne Fairhall |  |
| Eric Shea-Brown |  |
| Emily Sylwestrak |  |
| Andy Shih |  |
| Todd Woodward |  |
| Jeffrey LeDue | 1ffeRR4AAAAJ |
| Ilker Hacihaliloglu | dA7G64kAAAAJ |
| Randy Mcintosh | Ep3N640AAAAJ |
| Purang Abolmaesumi | gKZS5-IAAAAJ |
| Vesna Sossi | 5VQTWXgAAAAJ |

**Supplementary Table 2.** UBC Dynamic Brain Circuits Cluster author list. This is an example of the table required as input to the ScholarScraper notebook (as CSV). Note that the list contains one column for author names and another column for Scholar IDs which are both supported as inputs to the ScholarScraper notebook. Scholar IDs will be prioritized in the search function over the author name itself.

|  | **Faculty of Medicine** | **Faculty of Applied Science** | **Faculty of Science** | **Faculty of Arts** | **Not UBC** |
| --- | --- | --- | --- | --- | --- |
| **Tim H. Murphy** | Tim H. Murphy | |  |  |  |
| **Annie Ciernia** | |  | Annie Ciernia | |  |
| **Brian MacVicar** | Brian MacVicar | |  |  |  |
| **Fidel Vila-Rodriguez** | Fidel Vila-Rodriguez | |  |  |  |
| **Shernaz Bamji** | Shernaz Bamji | |  |  |  |
| **Lara Boyd** | Lara Boyd |  |  |  |  |
| **Paul Pavlidis** | Paul Pavlidis | |  |  |  |
| **Martin McKeown** | Martin McKeown | |  |  |  |
| **A Jon Stoessl** | A Jon Stoessl | |  |  |  |
| **Peter Cripton** | | Peter Cripton | |  |  |
| **Jason Snyder** | |  |  | Jason Snyder | |
| **Wolfram Tetzlaff** | |  | Wolfram Tetzlaff | |  |
| **Anthony Phillips** | Anthony Phillips | |  |  |  |
| **Catharine Winstanley** | |  |  | Catharine Winstanley | |
| **Yu Tian Wang** | Yu Tian Wang | |  |  |  |
| **Jeremy Seamans** | Jeremy Seamans | |  |  |  |
| **Terry Snutch** | |  | Terry Snutch | |  |
| **Ian Mackenzie** | Ian Mackenzie | |  |  |  |
| **Lynn Raymond** | Lynn Raymond | |  |  |  |
| **Kurt Haas** | Kurt Haas |  |  |  |  |
| **Mark Cembrowski** | Mark Cembrowski | |  |  |  |
| **Fabio Rossi** |  | Fabio Rossi |  |  |  |
| **Jane Roskams** | |  | Jane Roskams | |  |
| **Catharine Rankin** | |  |  | Catharine Rankin | |
| **Michael Gordon** | |  | Michael Gordon | |  |
| **Leonid Sigal** |  |  | Leonid Sigal |  |  |
| **Z. Jane Wang** | | Z. Jane Wang | |  |  |
| **Peyman Servati** | | Peyman Servati | |  |  |
| **Liisa Galea** |  |  |  | Liisa Galea |  |
| **Sophia Frangou** | Sophia Frangou | |  |  |  |
| **Silke Cresswell** | Silke Cresswell | |  |  |  |
| **Helge Rhodin** | |  | Helge Rhodin | |  |
| **Manu Madhav** | | Manu Madhav | |  |  |
| **Brian D. Fisher** | |  |  |  | Brian D. Fisher |
| **Leigh Anne Swayne** | |  |  |  | Leigh Anne Swayne |
| **Craig E. Brown** | |  |  |  | Craig E. Brown |
| **Adrienne Fairhall** | |  |  |  | Adrienne Fairhall |
| **Eric Shea-Brown** | |  |  |  | Eric Shea-Brown |
| **Emily Sylwestrak** | |  |  |  | Emily Sylwestrak |
| **Andy Shih** |  |  |  |  | Andy Shih |
| **Todd Woodward** | Todd Woodward | |  |  |  |
| **Jeffrey LeDue** | Jeffrey LeDue | |  |  |  |
| **Ilker Hacihaliloglu** | Ilker Hacihaliloglu | |  |  |  |
| **Randy Mcintosh** | |  |  |  | Randy Mcintosh |
| **Purang Abolmaesumi** | | Purang Abolmaesumi | |  |  |
| **Vesna Sossi** |  |  | Vesna Sossi |  |  |

**Supplementary Table 3.** UBC Dynamic Brain Circuits Cluster grouped author list. This is an example of the table required as input to the GroupedCollabs notebook (as CSV). Note that each group is represented by its own column, and each member appears in one group.

| **Name** | **Document Count** | **Cited by** | **Affiliation** | **Warning** |
| --- | --- | --- | --- | --- |
| **Tim H. Murphy** | 238 | 21285 | University of British Columbia |  |
| **Annie Ciernia** | 44 | 1986 | University of British Columbia, Vancouver | |
| **Brian MacVicar** | 218 | 22341 | Professor, University of British Columbia | |
| **Fidel Vila-Rodriguez** | 264 | 5786 | Assistant Professor; University of British Columbia | |
| **Shernaz Bamji** | 61 | 5352 | Professor, University of British Columbia | |
| **Lara Boyd** | 361 | 15313 | Professor, University of British Columbia | |
| **Paul Pavlidis** | 245 | 17772 | Professor of Psychiatry, University of British Columbia | |
| **Martin McKeown** | 370 | 16603 | Professor and Head, Division of Neurology, University of British Columbia | |
| **A Jon Stoessl** | 449 | 30910 | University of British Columbia |  |
| **Peter Cripton** | 284 | 7762 | Professor of Biomedical Engineering, University of British Columbia | |
| **Jason Snyder** | 40 | 6060 | Associate Professor, University of British Columbia | |
| **Wolfram Tetzlaff** | 315 | 24596 | Professor and Principal Investigator ICORD (International Collaboration on Repair Discoveries |  |
| **Anthony Phillips** | 428 | 37205 | University of British Columbia |  |
| **Catharine Winstanley** | 184 | 11301 | University of British Columbia |  |
| **Yu Tian Wang** | 200 | 42149 | University of British Columbia |  |
| **Jeremy Seamans** | 107 | 15820 | University of British Columbia |  |
| **Terry Snutch** | 462 | 34265 | University of British Columbia |  |
| **Ian Mackenzie** | 498 | 59289 | Division of Neuropathology, Department of Pathology and Lab Medicine, University of British Columbia | |
| **Lynn Raymond** | 171 | 19861 | Professor of Psychiatry, University of British Columbia, Huntington Study Group, Predict-HD | |
| **Kurt Haas** | 74 | 3714 | Full Professor of Neuroscience, University of British Columbia | |
| **Mark Cembrowski** | 35 | 2022 | Assistant Professor, University of British Columbia | |
| **Fabio Rossi** | 140 | 17022 | Professor of Medical Genetics, University of British Columbia | |
| **Jane Roskams** | 68 | 6142 | University of British Columbia/Washington | |
| **Catharine Rankin** | 209 | 7581 | University of British Columbia |  |
| **Michael Gordon** | 34 | 4120 | University of British Columbia |  |
| **Leonid Sigal** | 229 | 13421 | Professor, University of British Columbia | |
| **Z. Jane Wang** | 449 | 13906 | Professor of Electrical and Computer Engineering Dept., University of British Columbia, Canada | |
| **Peyman Servati** | 294 | 9534 | Electrical and Computer Engineering, University of British Columbia | |
| **Liisa Galea** | 297 | 22482 | Treliving Family Chair in Women's Mental Health, Senior Scientist, Centre for Addiction and Mental |  |
| **Sophia Frangou** | 638 | 26803 | Icahn School of Medicine at Mount SInai |  |
| **Silke Cresswell** | 98 | 4416 | Associate Professor, University of British Columbia | |
| **Helge Rhodin** | 83 | 4681 | Assistant Professor at UBC |  |
| **Manu Madhav** | 28 | 418 | Assistant Professor, University of British Columbia | |
| **Brian D. Fisher** | 187 | 3543 | Professor of Interactive Arts and Technology, Simon Fraser University | |
| **Leigh Anne Swayne** | 65 | 2041 | Professor, University of Victoria |  |
| **Craig E. Brown** | 39 | 2969 | University of Victoria, University of British Columbia | |
| **Adrienne Fairhall** | 119 | 5947 | Professor of Physiology and Biophysics, University of Washington | |
| **Eric Shea-Brown** | 197 | 7885 | Applied Mathematics, University of Washington | |
| **Emily Sylwestrak** | 16 | 1885 | University of Oregon | Affiliation does not match! |
| **Andy Shih** | 87 | 8051 | Seattle Children's Research Institute & University of Washington | |
| **Todd Woodward** | 296 | 13844 | Professor, University of British Columbia | |
| **Jeffrey LeDue** | 71 | 2633 | University of British Columbia |  |
| **Ilker Hacihaliloglu** | 116 | 2610 | Department of Radiology, Department of Medicine, University of British Columbia | |
| **Randy Mcintosh** | 474 | 47929 | Professor, Simon Fraser University | |
| **Purang Abolmaesumi** | 462 | 9873 | Department of Electrical and Computer Engineering, University of British Columbia, V6T 1Z4 | |
| **Vesna Sossi** | 500 | 18384 | Unknown affiliation |  |

**Supplementary Table 4.** Google Scholar Author data table. This is a portion of the table created by the ScholarScraper notebook as output (as CSV). Affiliations were flagged if they did not match one of: University of British Columbia, Simon Fraser University, University of Victoria, or University of Washington. The full version of this table will also contain the list of publication titles and coauthors.

| **Name** | **Coauthors** |
| --- | --- |
| **Tim H. Murphy** | {'Andy Shih': 9, 'Lynn Raymond': 18, 'Craig E. Brown': 6, 'Jeffrey LeDue': 40, 'Terry Snutch': 1, 'Yu Tian Wang': 4, 'Brian MacVicar': 3, 'Wolfram Tetzlaff': 2, 'Helge Rhodin': 2, 'Peter Cripton': 1, 'Mark Cembrowski': 1} |
| **Annie Ciernia** | {'Brian MacVicar': 1} |
| **Brian MacVicar** | {'Tim H. Murphy': 3, 'Jeffrey LeDue': 13, 'Annie Ciernia': 1, 'Terry Snutch': 9, 'Anthony Phillips': 1, 'Shernaz Bamji': 1, 'Yu Tian Wang': 1, 'Silke Cresswell': 1, 'Leigh Anne Swayne': 1, 'Fabio Rossi': 1, 'Ian Mackenzie': 1} |
| **Fidel Vila-Rodriguez** | {'Z. Jane Wang': 1, 'Sophia Frangou': 6, 'Jason Snyder': 2, 'Silke Cresswell': 1, 'Ian Mackenzie': 1, 'Todd Woodward': 1} |
| **Shernaz Bamji** | {'Brian MacVicar': 1, 'Ian Mackenzie': 1, 'Lynn Raymond': 2, 'Terry Snutch': 1, 'Paul Pavlidis': 2, 'Kurt Haas': 6, 'Catharine Rankin': 2, 'Anthony Phillips': 1} |
| **Lara Boyd** | {'Martin McKeown': 2, 'A Jon Stoessl': 2, 'Silke Cresswell': 1, 'Vesna Sossi': 2, 'Todd Woodward': 3, 'Liisa Galea': 1} |
| **Paul Pavlidis** | {'Shernaz Bamji': 2, 'Kurt Haas': 6, 'Catharine Rankin': 6, 'Terry Snutch': 1, 'Sophia Frangou': 1, 'Vesna Sossi': 1} |
| **Martin McKeown** | {'Lara Boyd': 2, 'A Jon Stoessl': 29, 'Silke Cresswell': 33, 'Vesna Sossi': 21, 'Z. Jane Wang': 72, 'Brian D. Fisher': 1, 'Peyman Servati': 1, 'Catharine Winstanley': 1} |
| **A Jon Stoessl** | {'Lara Boyd': 2, 'Martin McKeown': 29, 'Silke Cresswell': 25, 'Vesna Sossi': 134, 'Catharine Winstanley': 3, 'Anthony Phillips': 2, 'Ian Mackenzie': 6, 'Terry Snutch': 1} |
| **Peter Cripton** | {'Tim H. Murphy': 1, 'Mark Cembrowski': 1, 'Jeffrey LeDue': 1, 'Wolfram Tetzlaff': 14, 'Catharine Winstanley': 1} |
| **Jason Snyder** | {'Fidel Vila-Rodriguez': 2} |
| **Wolfram Tetzlaff** | {'Tim H. Murphy': 2, 'Peter Cripton': 14, 'Fabio Rossi': 1, 'Jane Roskams': 9, 'Silke Cresswell': 1} |
| **Anthony Phillips** | {'Brian MacVicar': 1, 'Shernaz Bamji': 1, 'A Jon Stoessl': 2, 'Vesna Sossi': 2, 'Jeremy Seamans': 15, 'Yu Tian Wang': 11, 'Todd Woodward': 1, 'Catharine Winstanley': 1, 'Terry Snutch': 3} |
| **Catharine Winstanley** | {'Martin McKeown': 1, 'A Jon Stoessl': 3, 'Silke Cresswell': 1, 'Peter Cripton': 1, 'Anthony Phillips': 1, 'Vesna Sossi': 3, 'Jeremy Seamans': 1} |
| **Yu Tian Wang** | {'Tim H. Murphy': 4, 'Jeffrey LeDue': 2, 'Brian MacVicar': 1, 'Anthony Phillips': 11, 'Jeremy Seamans': 1, 'Lynn Raymond': 3, 'Catharine Rankin': 1} |
| **Jeremy Seamans** | {'Anthony Phillips': 15, 'Yu Tian Wang': 1, 'Catharine Winstanley': 1, 'Todd Woodward': 1} |
| **Terry Snutch** | {'Tim H. Murphy': 1, 'Brian MacVicar': 9, 'Jeffrey LeDue': 3, 'Ian Mackenzie': 1, 'Shernaz Bamji': 1, 'Paul Pavlidis': 1, 'A Jon Stoessl': 1, 'Anthony Phillips': 3, 'Catharine Rankin': 1} |
| **Ian Mackenzie** | {'Brian MacVicar': 1, 'Terry Snutch': 1, 'Fidel Vila-Rodriguez': 1, 'Shernaz Bamji': 1, 'Lynn Raymond': 3, 'A Jon Stoessl': 6, 'Vesna Sossi': 6} |
| **Lynn Raymond** | {'Tim H. Murphy': 18, 'Shernaz Bamji': 2, 'Ian Mackenzie': 3, 'Yu Tian Wang': 3, 'Jeffrey LeDue': 2} |
| **Kurt Haas** | {'Shernaz Bamji': 6, 'Paul Pavlidis': 6, 'Catharine Rankin': 5} |
| **Mark Cembrowski** | {'Tim H. Murphy': 1, 'Peter Cripton': 1, 'Jeffrey LeDue': 1} |
| **Fabio Rossi** | {'Brian MacVicar': 1, 'Wolfram Tetzlaff': 1} |
| **Jane Roskams** | {'Wolfram Tetzlaff': 9} |
| **Catharine Rankin** | {'Shernaz Bamji': 2, 'Paul Pavlidis': 6, 'Kurt Haas': 5, 'Yu Tian Wang': 1, 'Terry Snutch': 1, 'Liisa Galea': 1} |
| **Michael Gordon** | |
| **Leonid Sigal** | {'Helge Rhodin': 1} |
| **Z. Jane Wang** | {'Fidel Vila-Rodriguez': 1, 'Martin McKeown': 72, 'Silke Cresswell': 4, 'Peyman Servati': 2, 'Helge Rhodin': 1} |
| **Peyman Servati** | {'Martin McKeown': 1, 'Z. Jane Wang': 2} |
| **Liisa Galea** | {'Lara Boyd': 1, 'Catharine Rankin': 1, 'Sophia Frangou': 1} |
| **Sophia Frangou** | {'Fidel Vila-Rodriguez': 6, 'Paul Pavlidis': 1, 'Vesna Sossi': 1, 'Liisa Galea': 1, 'Eric Shea-Brown': 1, 'Randy Mcintosh': 3} |
| **Silke Cresswell** | {'Brian MacVicar': 1, 'Fidel Vila-Rodriguez': 1, 'Lara Boyd': 1, 'Martin McKeown': 33, 'A Jon Stoessl': 25, 'Vesna Sossi': 15, 'Z. Jane Wang': 4, 'Catharine Winstanley': 1, 'Wolfram Tetzlaff': 1} |
| **Helge Rhodin** | {'Tim H. Murphy': 2, 'Jeffrey LeDue': 1, 'Leonid Sigal': 1, 'Z. Jane Wang': 1} |
| **Manu Madhav** | |
| **Brian D. Fisher** | {'Martin McKeown': 1} |
| **Leigh Anne Swayne** | {'Brian MacVicar': 1, 'Craig E. Brown': 1} |
| **Craig E. Brown** | {'Tim H. Murphy': 6, 'Leigh Anne Swayne': 1} |
| **Adrienne Fairhall** | {'Eric Shea-Brown': 3} |
| **Eric Shea-Brown** | {'Sophia Frangou': 1, 'Adrienne Fairhall': 3} |
| **Emily Sylwestrak** | |
| **Andy Shih** | {'Tim H. Murphy': 9} |
| **Todd Woodward** | {'Fidel Vila-Rodriguez': 1, 'Lara Boyd': 3, 'Anthony Phillips': 1, 'Jeremy Seamans': 1, 'Vesna Sossi': 1} |
| **Jeffrey LeDue** | {'Tim H. Murphy': 40, 'Brian MacVicar': 13, 'Yu Tian Wang': 2, 'Helge Rhodin': 1, 'Peter Cripton': 1, 'Mark Cembrowski': 1, 'Terry Snutch': 3, 'Lynn Raymond': 2} |
| **Ilker Hacihaliloglu** | {'Purang Abolmaesumi': 11} |
| **Randy Mcintosh** | {'Sophia Frangou': 3} |
| **Purang Abolmaesumi** | {'Ilker Hacihaliloglu': 11} |
| **Vesna Sossi** | {'Lara Boyd': 2, 'Martin McKeown': 21, 'A Jon Stoessl': 134, 'Silke Cresswell': 15, 'Paul Pavlidis': 1, 'Sophia Frangou': 1, 'Anthony Phillips': 2, 'Ian Mackenzie': 6, 'Catharine Winstanley': 3, 'Todd Woodward': 1} |

**Supplementary Table 5.** Google Scholar Coauthor data table. This is a portion of the table created by the ScholarScraper notebook as output (as CSV). These two columns are used as input for the ScholarCollab and GroupedCollabs notebooks to produce the collaboration diagrams (Fig. 4a and Fig. 5a).

| **Name** | **Coauthors** |
| --- | --- |
| **Tim H. Murphy** | {'Lynn Raymond': 17, 'Jeffrey LeDue': 25, 'Helge Rhodin': 2, 'Brian MacVicar': 2, 'Yu Tian Wang': 4, 'Wolfram Tetzlaff': 2, 'Craig E. Brown': 6, 'Andy Shih': 9, 'Terry Snutch': 1} |
| **Annie Ciernia** |  |
| **Brian MacVicar** | {'Tim H. Murphy': 2, 'Jeffrey LeDue': 10, 'Silke Cresswell': 1, 'Terry Snutch': 5, 'Leigh Anne Swayne': 1, 'Shernaz Bamji': 1, 'Yu Tian Wang': 2, 'Anthony Phillips': 1} |
| **Fidel Vila-Rodriguez** | {'Sophia Frangou': 5, 'Ian Mackenzie': 1, 'Jason Snyder': 2, 'Silke Cresswell': 1, 'Z. Jane Wang': 1} |
| **Shernaz Bamji** | {'Brian MacVicar': 1, 'Yu Tian Wang': 1, 'Kurt Haas': 3, 'Lynn Raymond': 2, 'Paul Pavlidis': 1, 'Catharine Rankin': 1, 'Terry Snutch': 1, 'Anthony Phillips': 1, 'Ian Mackenzie': 1} |
| **Lara Boyd** | {'Liisa Galea': 1, 'Martin McKeown': 2, 'A Jon Stoessl': 1, 'Silke Cresswell': 1, 'Vesna Sossi': 1, 'Todd Woodward': 2} |
| **Paul Pavlidis** | {'Shernaz Bamji': 1, 'Kurt Haas': 3, 'Catharine Rankin': 3, 'Jeffrey LeDue': 1} |
| **Martin McKeown** | {'Lara Boyd': 2, 'A Jon Stoessl': 16, 'Silke Cresswell': 14, 'Vesna Sossi': 11, 'Z. Jane Wang': 82, 'Peyman Servati': 1} |
| **A Jon Stoessl** | {'Lara Boyd': 1, 'Martin McKeown': 16, 'Silke Cresswell': 14, 'Vesna Sossi': 73, 'Ian Mackenzie': 2, 'Anthony Phillips': 2, 'Terry Snutch': 1} |
| **Peter Cripton** | {'Wolfram Tetzlaff': 6} |
| **Jason Snyder** | {'Fidel Vila-Rodriguez': 2} |
| **Wolfram Tetzlaff** | {'Tim H. Murphy': 2, 'Peter Cripton': 6, 'Jane Roskams': 9, 'Fabio Rossi': 1} |
| **Anthony Phillips** | {'Brian MacVicar': 1, 'Shernaz Bamji': 1, 'A Jon Stoessl': 2, 'Vesna Sossi': 2, 'Terry Snutch': 3, 'Jeremy Seamans': 15, 'Yu Tian Wang': 14, 'Z. Jane Wang': 1, 'Todd Woodward': 1} |
| **Catharine Winstanley** | {'Vesna Sossi': 1} |
| **Yu Tian Wang** | {'Tim H. Murphy': 4, 'Jeffrey LeDue': 3, 'Brian MacVicar': 2, 'Shernaz Bamji': 1, 'Anthony Phillips': 14, 'Jeremy Seamans': 1, 'Lynn Raymond': 2, 'Ian Mackenzie': 1, 'Catharine Rankin': 1} |
| **Jeremy Seamans** | {'Anthony Phillips': 15, 'Yu Tian Wang': 1, 'Todd Woodward': 1} |
| **Terry Snutch** | {'Tim H. Murphy': 1, 'Brian MacVicar': 5, 'Jeffrey LeDue': 2, 'Shernaz Bamji': 1, 'A Jon Stoessl': 1, 'Anthony Phillips': 3} |
| **Ian Mackenzie** | {'Fidel Vila-Rodriguez': 1, 'Shernaz Bamji': 1, 'Lynn Raymond': 2, 'A Jon Stoessl': 2, 'Vesna Sossi': 2, 'Yu Tian Wang': 1} |
| **Lynn Raymond** | {'Tim H. Murphy': 17, 'Shernaz Bamji': 2, 'Ian Mackenzie': 2, 'Yu Tian Wang': 2, 'Jeffrey LeDue': 1} |
| **Kurt Haas** | {'Shernaz Bamji': 3, 'Paul Pavlidis': 3, 'Catharine Rankin': 2} |
| **Mark Cembrowski** |  |
| **Fabio Rossi** | {'Wolfram Tetzlaff': 1} |
| **Jane Roskams** | {'Wolfram Tetzlaff': 9} |
| **Catharine Rankin** | {'Shernaz Bamji': 1, 'Paul Pavlidis': 3, 'Kurt Haas': 2, 'Yu Tian Wang': 1} |
| **Michael Gordon** |  |
| **Leonid Sigal** | {'Helge Rhodin': 1} |
| **Z. Jane Wang** | {'Fidel Vila-Rodriguez': 1, 'Martin McKeown': 82, 'Silke Cresswell': 2, 'Anthony Phillips': 1, 'Helge Rhodin': 2, 'Liisa Galea': 1, 'Brian D. Fisher': 1, 'Purang Abolmaesumi': 1, 'Peyman Servati': 1} |
| **Peyman Servati** | {'Martin McKeown': 1, 'Z. Jane Wang': 1} |
| **Liisa Galea** | {'Lara Boyd': 1, 'Z. Jane Wang': 1, 'Brian D. Fisher': 1, 'Purang Abolmaesumi': 1, 'Sophia Frangou': 1} |
| **Sophia Frangou** | {'Fidel Vila-Rodriguez': 5, 'Liisa Galea': 1} |
| **Silke Cresswell** | {'Brian MacVicar': 1, 'Fidel Vila-Rodriguez': 1, 'Lara Boyd': 1, 'Martin McKeown': 14, 'A Jon Stoessl': 14, 'Vesna Sossi': 6, 'Z. Jane Wang': 2} |
| **Helge Rhodin** | {'Tim H. Murphy': 2, 'Jeffrey LeDue': 1, 'Leonid Sigal': 1, 'Z. Jane Wang': 2} |
| **Manu Madhav** |  |
| **Brian D. Fisher** | {'Z. Jane Wang': 1, 'Liisa Galea': 1, 'Purang Abolmaesumi': 2} |
| **Leigh Anne Swayne** | {'Brian MacVicar': 1, 'Craig E. Brown': 1} |
| **Craig E. Brown** | {'Tim H. Murphy': 6, 'Leigh Anne Swayne': 1} |
| **Adrienne Fairhall** | {'Eric Shea-Brown': 3} |
| **Eric Shea-Brown** | {'Adrienne Fairhall': 3} |
| **Emily Sylwestrak** |  |
| **Andy Shih** | {'Tim H. Murphy': 9} |
| **Todd Woodward** | {'Lara Boyd': 2, 'Anthony Phillips': 1, 'Jeremy Seamans': 1, 'Vesna Sossi': 1} |
| **Jeffrey LeDue** | {'Tim H. Murphy': 25, 'Helge Rhodin': 1, 'Brian MacVicar': 10, 'Yu Tian Wang': 3, 'Terry Snutch': 2, 'Paul Pavlidis': 1, 'Lynn Raymond': 1} |
| **Ilker Hacihaliloglu** | {'Purang Abolmaesumi': 11} |
| **Randy Mcintosh** |  |
| **Purang Abolmaesumi** | {'Z. Jane Wang': 1, 'Liisa Galea': 1, 'Brian D. Fisher': 2, 'Ilker Hacihaliloglu': 11} |
| **Vesna Sossi** | {'Lara Boyd': 1, 'Martin McKeown': 11, 'A Jon Stoessl': 73, 'Silke Cresswell': 6, 'Ian Mackenzie': 2, 'Anthony Phillips': 2, 'Catharine Winstanley': 1, 'Todd Woodward': 1} |

**Supplementary Table 6.** SciVal Coauthor data table. This is a portion of the table created by the ScholarScraper notebook as output (as CSV). These two columns are used as input for the ScholarCollab and GroupedCollabs notebooks to produce the collaboration diagrams (Fig. 4b and Fig. 5b).

| **Folder** | **File** | **Short Description** | **Code Structure/Table Properties** |
| --- | --- | --- | --- |
| Main folder | ScholarScraper.ipynb | Python Jupyter Notebook used for scraping information. Outputs *ss_output_data.csv*. Described in Section 2.2. | 1. Install libraries 2. Modify names of input and output files 3. Load author names 4. Modify affiliations list 5. Scrape data for each author 6. Add co-authors to the rows 7. Write rows to output CSV file 8. Create barplot citations per year 9. Create collaboration heatmap |
|  | ScholarCollabs.ipynb | R Jupyter Notebook used for creating co-author chord diagrams with *ss_output_data.csv*. Described in Section 2.3. | 1. Install and load libraries 2. Import data file 3. Define title, colors, variables of the graph 4. Load in collaboration data 5. Tidy the dataframe 6. Set up the links 7. Assign a color to each investigator 8. Create the chord diagram |
|  | GroupedCollabs.ipynb | R Jupyter Notebook used for creating grouped co-author chord diagrams with *ss_output_data.csv* and *dbc_faculty_groups.csv*. Described in Section 2.3. | 1. Install libraries 2. Modify names of input and output files 3. Load author names 4. Modify affiliations list 5. Scrape data for each author 6. Add co-authors to the rows 7. Write rows to output CSV file 8. Create barplot citations per year 9. Create collaboration heatmap |
|  | *authorlist.csv* | A table of desired authors to scrape data for. The ScholarScraper notebook reads to search for author publications on GS, as detailed in Section 2.2 and Supplementary Table 2. | Columns: “Name”, “GSID”.  Each row represents an author. |
|  | *dbc_faculty_groups.csv* | A table which contains rows for each author and columns of the research group which each author belongs to. This file is read by the GroupedCollabs notebook, as described in Section 2.3. | Columns: “Faculty of Medicine”, “Faculty of Applied Science”, “Faculty of Science”, “Faculty of Arts”, “Not UBC”. Each row represents an author. |
| Output folder | *shared_pubs.csv* | A table with columns “Title” and “Authors”. This is generated by the ScholarScraper notebook. | Columns: “Title”, “Authors”.  Each row represents a shared publication found on all of the listed authors’ profiles. |
|  | *ss_output_data.csv* | ScholarScraper notebook outcome which describes the scraped information for each author. This is further described in Section 3.1. | Columns: “Name”, “Name on Profile”, “Scholar ID”, “Document Count”, “Cited by”, “Cited by 5 years”, “h-index”, “h-index 5 years”, “i10-index”, “i10-index 5 years”, “Publications”, “co-authors”, “Affiliation”, “Warning”.  Each row represents an author. |

**Supplementary Table 7.** Summary and Reference for Code and Files on GitHub.
